# Triage by ranking to support the curation of protein interactions

**DOI:** 10.1093/database/bax040

**Published:** 2017-06-11

**Authors:** Luc Mottin, Emilie Pasche, Julien Gobeill, Valentine Rech de Laval, Anne Gleizes, Pierre-André Michel, Amos Bairoch, Pascale Gaudet, Patrick Ruch

**Affiliations:** 1 Information Science Department, BiTeM Group, HES-SO/HEG Genève, 17 Rue de la Tambourine, Carouge CH-1227, Switzerland; 2 SIB Text Mining, Swiss Institute of Bioinformatics, 17 Rue de la Tambourine, Carouge CH-1227, Switzerland; 3 CALIPHO Group, Swiss Institute of Bioinformatics, 1 Rue Michel-Servet, Geneva CH-1206, Switzerland; 4 University of Geneva, Geneva

## Abstract

Today, molecular biology databases are the cornerstone of knowledge sharing for life and health sciences. The curation and maintenance of these resources are labour intensive. Although text mining is gaining impetus among curators, its integration in curation workflow has not yet been widely adopted. The Swiss Institute of Bioinformatics Text Mining and CALIPHO groups joined forces to design a new curation support system named nextA5. In this report, we explore the integration of novel triage services to support the curation of two types of biological data: protein–protein interactions (PPIs) and post-translational modifications (PTMs). The recognition of PPIs and PTMs poses a special challenge, as it not only requires the identification of biological entities (proteins or residues), but also that of particular relationships (e.g. binding or position). These relationships cannot be described with onto-terminological descriptors such as the Gene Ontology for molecular functions, which makes the triage task more challenging. Prioritizing papers for these tasks thus requires the development of different approaches. In this report, we propose a new method to prioritize articles containing information specific to PPIs and PTMs. The new resources (RESTful APIs, semantically annotated MEDLINE library) enrich the neXtA5 platform. We tuned the article prioritization model on a set of 100 proteins previously annotated by the CALIPHO group. The effectiveness of the triage service was tested with a dataset of 200 annotated proteins. We defined two sets of descriptors to support automatic triage: the first set to enrich for papers with PPI data, and the second for PTMs. All occurrences of these descriptors were marked-up in MEDLINE and indexed, thus constituting a semantically annotated version of MEDLINE. These annotations were then used to estimate the relevance of a particular article with respect to the chosen annotation type. This relevance score was combined with a local vector-space search engine to generate a ranked list of PMIDs. We also evaluated a query refinement strategy, which adds specific keywords (such as ‘binds’ or ‘interacts’) to the original query. Compared to PubMed, the search effectiveness of the nextA5 triage service is improved by 190% for the prioritization of papers with PPIs information and by 260% for papers with PTMs information. Combining advanced retrieval and query refinement strategies with automatically enriched MEDLINE contents is effective to improve triage in complex curation tasks such as the curation of protein PPIs and PTMs.

**Database URL:** http://candy.hesge.ch/nextA5

## Introduction

A large area of the research in biology aims to improve the understanding of the cell, and in particular, to investigate how the proteins from these cells collaborate together. Involved in numerous molecular processes, protein–protein interactions (PPIs) are deeply described in the literature (1–3). Post-translational modifications (PTMs) are typically defined as the alteration of a protein after its synthesis (4). These modifications often imply an enzymatic process such as the phosphorylation in which a phosphate is covalently attached to an amino acid side chain. Moreover, these modifications are important for the functional activity of cells by regulating their activity and interactions, among other mechanisms. Today, about 200 such modification types are known (5), but our study focuses on the PTMs that represent most of the ones we can find in the literature.

Current research in life sciences has become greatly sustained by the many knowledge bases available for the community. Curators play a key role to define the content and ensure the quality of the biomedical databases and to spotlight the major findings (6–8). Their mission consists of continuously collecting, verifying and annotating the literature using ontologies and other data sources. Although this process is accurate, it is also time-consuming and improved approaches have been proposed by the text-mining community.

Many text-mining methods have been applied to support the annotation of proteins, including the capture of information describing their normal or pathologic functions (9–18). Molecular functions, subcellular locations, biological processes and diseases are among the most frequent entities extracted by these methods. These methods are supported by a wealth of onto-terminological resources. One limitation with approaches, which depends on ontological resources and descriptor dictionaries, is that they do not support the extraction of protein–protein interactions, as the high combinatory nature of such data—virtually any protein can interact with any other protein or even protein-complex—makes it impossible to generate a controlled vocabulary that captures all the possible combinations. This problem is known as relationship extraction (19). Several experiments have been made to build relationships on top of a simple named-entity recognizer. Once some proteins are identified, the recognition of specific triggers [verbs such as *bind* or nouns such as *complex* (13–18)] can be used to build the interaction tuple. However, these methods, which attempt to replace the role of the curator by identifying each entity involved in the interaction, are relatively complex to tune so that success rates are relatively low even for a limited set of species. For example, at BioCreative II, the precision for the interaction pair extraction task was in a range from 5% to 39% with an average of 18% (20).

In (21), the authors estimate that about 7% of the curation time is assigned to the rejection of papers. From such a study, we assume that at least the same amount of time (so another ∼7%) percent is needed to select relevant papers. Altogether, we suggest that about 15% of the curation effort is spent on triage. By exclusion, we can thus consider that 85% of the effort relates to other curation tasks, including reading (e.g. text, images, tables…) and normalization (e.g. choosing unique accession number or descriptors in a controlled vocabulary); see (22) for the presentation of a sequential curation workflow. So, we hypothesize that by improving the search effectiveness by +100%, we can save about 7–8% of the productivity of a curation team. Linearly, an improvement of +200% would result in speeding up curation by about 10%.

In addition to delivering a better triage, the search engines we explore in this report will also be more specialized and address more specific curation needs. Although they may not perform better than existing ones (e.g. PubMed, EBIMed, EAGLi…) as general-purpose search tools, we expect they will be optimal to rank the literature given a particular annotation task. In previous works, we presented successful examples of customized engines powered with onto-terminological resources (12) to support the annotation of diseases or gene functions; today, we extend the work to support the curation of data type involving relationships.

One of our objectives is to provide professional curators with documents likely to contain information relevant for the curation of PPIs and PTMs. We started working with neXtProt, a database maintained by the Swiss Institute of Bioinformatics (SIB) (23) and we focus our attention on the triage task. Ultimately, interacting entities (proteins, interaction types, methods to predict interactions…) will be proposed for validation by the system, but first, we want to reduce the search burden by simplifying the paper selection step (24, 25). The selection process itself is seen as an interactive process, piloted by the curator. The most relevant articles are first ranked based on the content of their abstracts. Once they have been selected by the curator, the system triggers some dedicated information extraction module with the creation and display of triplets {subject–relation–object}. These triplets will finally be validated—rejected or modified—by the curator.

In parallel, we also explore a related problem: the curation of PTMs. The idea is to test the scalability and the generalization power of the triage system to address the identification of papers dealing with a different type of biological relationship. Our evaluations are made on 16 types of PTM, but previous work showed that virtually all PTMs are expressed with the same semantic model (26): the protein (subject), the substrate (object) and the exact position (location) affected by the PTM. Finally, we also describe in this report how the services are embedded in the curation workflow of neXtProt, within the BioEditor curation tool (27).

## Materials and methods

### Investigated molecular processes

Regarding the terminological resources used in our experiments, the Proteomics Standards Initiative provides community standards for data representation in proteomics (28–30), which have also been applied to PPIs. A subset of a few verbs and assays was also selected. From this group, we generated a list of morphological variants (or stems) such as *phosphorylate*—which is able to cover strings such as *phosphorylated, phosphorylates, phosphorylating and phosphorylation*. Thus, we end up with a very specific vocabulary, which contains 23 concepts: 14 stemmed interaction terms together with the 9 most annotated experimental methods found in the neXtProt database. This thesaurus is presented as supplementary material in file 1.

This study focuses on 16 different PTMs, which are also among the most frequent: phosphorylation, methylation, dephosphorylation, glycosylation, nitrosylation, palmitoylation, deubiquitination, polyADP-ribosylation, acetylation, desumoylation, myristoylation, deacetylation, farnesylation, ubiquitination, sumoylation and protein cleavages.

### BioMed and neXtA^5^

To initiate the literature search, we used BioMed, a fully synchronized mirror of MEDLINE and PMC. BioMed is stratified into temporal bins so that a user can query specific temporal ranges. Our experiments are made on a bin containing about 16 million PubMed abstracts, i.e. all MEDLINE from 1990 until today. The content of BioMed is enriched by several millions of automatic annotations from several ontologies (MeSH, NCI Thesaurus, Gene Ontology, etc.), generated by neXtA^5^ [12]. All BioMed contents, including the annotations, are freely available. Specific subsets are also pushed on a regular basis to enrich the content of Europe PMC (31, 32). To support our experiments, BioMed was enriched in order to annotate also interaction concepts. The sentences and offset positions where the concepts are found are also recorded, and the database is regularly updated.

More than a database, BioMed is a digital library resource developed by SIB Text Mining to primarily serve SIB internal needs. The library integrates the Terrier search engine (33). The search effectiveness of the IR module has been demonstrated during various competitions (34, 35) with default scoring functions derived from the Okapi BM25 and deviation from randomness weighting schemas (36, 37).

At curation time, neXtA^5^ queries the IR module from BioMed and also fetches the associated annotations. The initial search step, known as ‘vector space model’, determines the relevance of documents with respect to the initial query. Thus, if the initial query is a protein, then the scoring function will rank papers that contain several occurrences of the protein. Compared to the default Boolean function of other engines, which rank papers by chronological order (most recent first), this is an advantage. In complement, and to improve recall, synonyms or keywords may be added to the initial query. The first result of the ranking is calculated based on the density of the query terms in each document, but the score is then combined with other parameters in a linear combination specifically tuned for each axis (PPIs or PTMs).

The system also proposes a more traditional research mode based on the PubMed Boolean model (38). In that case, the articles are ranked by PubMed, but the results are enriched with the annotations stored in BioMed.

### Fusion of search scores

The combination of results provided by different engines, which is known to improve search effectiveness in a large set of situations (39–41), is also tested in our environment. The new ranking is based on the linear combination of two components: the vector-space search engine and the ranking provided by computing the density of either PPIs or PTMs descriptors (i.e. the total of different PTMs or PPIs concepts occurring in a document). Then, to obtain the optimal tuning, we assessed several combinations of parameters by varying their respective weights. The tuning experiments generated the following formulae for PPIs and PTMs:

Protein–Protein Interactions:

Linear combination=0.9×search engine score+1.5×∑distinct descriptor

Post-Translational Modifications:

Linear combination=0.9×search engine score+1.7×∑distinct descriptor

This optimized selection of parameters was derived by trial and errors by testing some variant combinations based on the well-known term frequency (TF) inverse document frequency (IDF) model. In scientific publications, concepts are often repeated, and TF (i.e. the number of times a given concepts occurs in a given document) is a commonly used measure of information retrieval systems. However, results showed that using TF in combination with the features in the aforementioned formulae does not improve the retrieval effectiveness of the triage method. The IDF is taken in account in the weighting schema computed by the BioMed search.

### Query refinement

In a second set of experiments, we evaluate the effect of specific keywords (added at query time) on the retrieval effectiveness of the model. Terms such as ‘associates’, ‘binds’, ‘interacts’, ‘phosphorylates’ and their combinations are added to expand the original queries. We assessed the impact of these terms on the retrieval effectiveness of the triage engine for both PPIs and PTMs. A new linear combination is generated, which uses two new statistical measures: the TF of a descriptor and its length measured in characters. The length of the descriptors can indeed be seen as an approximation of its specificity (42–44). The comprehensive list of keywords tested, as well as the associated results, is provided as supplementary material in file 2. The best improvements were observed with the following combinations:

Protein–Protein Interactions: keyword =  ‘binds +  interacts  + associates’

$$\text{Linear}\;\text{combination}=1.0\times \text{search}\;\text{engine}\;\text{score}+0.1\times \sum_{\text{descriptor}}\;\text{log}(1+\text{descriptor}\;\text{length}\times \text{term}\;\text{frequency}\;\text{of}\;\text{descriptor})\;$$

Post-Translational Modifications: keyword =  ‘phosph or y lates’

Linear combination=1.4×search engine score+1.3×∑distinct descriptor

Interestingly, the TF parameter, which did not improve our results in the previous experiments, has now a positive impact of about +10% for retrieving documents relevant to curate PPIs. In contrast, the number of unique concepts does not improve the selection of papers, suggesting that the TF and the number of unique descriptors capture similar information. The importance of the keyword ‘phosphorylates’ for PTMs reflects the high number of phosphorylations in the PTM data set, which account from about 95% of all PTMs, and using other keywords does not result in any comparable improvements.

## Evaluation

This evaluation focused on the pertinence of the documents retrieved, and the precision is the reference metric established for such task; see the Text Retrieval Conference (TREC) (24, 45).

### Datasets

In the course of the annotation of 300 human protein kinases, curators captured 4044 binary interactions and 5862 PTMs. The PMIDs selected by our curators for the sake of annotation are used as gold standard (the so-called relevance judgements or QREL in the TREC jargon) in our experiments. Within these data, we used a subset of 100 kinases to tune the system with 1100 annotations for the PPIs and 1933 for the PTM axis. The rest of the data were used for the evaluation of the new triage methods. The 300 kinases are provided as supplementary material in file 3, whereas the PPIs and PTMs QREL are, respectively, presented as supplementary material in files 4-a and 4-b.

As PubMed is the traditional source of information used by curators (and researchers), we adopted it as the baseline. For each of the 100 kinases subsets, we consider the top 1000 PMIDs returned by the engines. PMIDs published outside the temporal boundaries of our benchmarks (i.e. papers published after December 2013) are simply discarded. Indeed, all search strategies should in principle be equally affected by such a temporal drift; however, we thought that not filtering out by dates could have been slightly detrimental for PubMed, because publication dates play a significant role in the ranking of the results by the NLM engine. PubMed is queried via the Boolean mode (NCBI E-utilities) using the relevance sort option. Reviews and retracted publications are excluded from the results of all search results because they are not used by biocurators. 

### Metrics

Since we aim at facilitating the curators work, we try to model the curation workflow, and, in particular, the search behaviour of curators. Retrieval effectiveness is assessed using different metrics such as mean average precision (MAP), which averages the precision at different points of recall (precision at 5 documents, 10 documents, etc.). Recall is usually ignored because in a large document collection, it is impossible to list of relevant papers. Similarly, a curator does not try to keep track of all relevant papers for a given protein or gene product, instead the curator wants to record all information about a given gene product. If a particular statement or fact about a protein is found in several articles, the curator will capture the statement but rarely records all papers containing the statement.

For a human agent, who interacts with a search engine, the top ranked documents are clearly more useful than documents found at lower ranks. In this context, we pay special attention to the precision of the top-returned document, the so-called mean reciprocal rank or P0.

As detailed in Table 1, the subset of documents relevant for the 100 kinases contains 1100 and 1933 PMIDs, respectively, for PPIs and PTMs. Between 29 782 and 87 998 PMIDs are then returned by the different search engines we are comparing, which means that the percentage of true positive results is relatively low.

**Table 1.** Document_distribution

***A***	**PUBMED**	**BIOMED**	**QUERY EXPANSION**
**# OF RETRIEVED DOCUMENTS**	33 408	29 782	84 629
**# OF RELEVANT DOCUMENTS**	1100	1100	1100
**# OF RELEVANT_RETRIEVED DOCUMENTS**	469	504	469

***B***	**PUBMED**	**BIOMED**	**QUERY EXPANSION**
**# OF RETRIEVED DOCUMENTS**	33 408	29 782	87 998
**# OF RELEVANT DOCUMENTS**	1933	1933	1933
**# OF RELEVANT_RETRIEVED DOCUMENTS**	607	584	689


Distribution of the documents retrieved by the different systems during A—the PPIs evaluation, and B—the PTMs evaluation. The query expansions mentioned in table A and B, respectively, relate to the keywords ‘phosphorylate’ and ‘binds + interacts + association’

## Results

The first module evaluated is the vector space search engine from BioMed. We immediately observe a gain at P0, i.e. the chance to find a relevant document at first rank, compared to PubMed. With 0.14 for the PPIs and 0.16 for the PTMs, BioMed improves the baseline by, respectively, +34% and +57%. Regarding simple search (the so-called *ad hoc* by TREC), the use of a vector-space model compared to PubMed is already effective.

In a second step, we measure the effectiveness of the linear combination of the BioMed ranking and the ranking provided based on the density of descriptors. The second strategy of triage outperforms both PubMed and BioMed, with a P0 value at 0.27 for the PPIs and 0.28 for the PTMs. The improvement already confirms the advantage of developing distinct methods to explore these two particular axes. Furthermore, using the query refinement strategy brings some modest yet significant improvements, with a gain of 11% (PPIs) and 30% (PTM).

**Figure 1.**
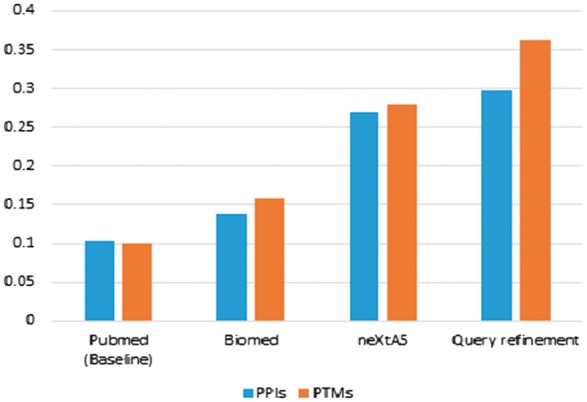
Comparison of the precision at P0 for the PPIs and PTMs ranking task by using PubMed versus BioMed, neXtA^5^ and neXtA^5^ augmented with the query refinement approach.

The overall improvement compared to PubMed reaches +191% and  +261% for PPIs and PTMs, respectively (Table 2, Figure 1). In other words, for PTMs, PubMed returns one relevant article out of 10, whereas neXtA^5^ returns one relevant article out of three at the top of the list.

Finally, regarding PTMs, the impact of the single keyword ‘phosphorylate’ (or its stemmed form) suggests that designing a strategy for each PTM could result in some significant improvement of the triage effectiveness. The improvement is more modest but relatively similar for PPIs by using a combination of keywords.

## Implementation

Currently, we developed and deployed two implementations of the triage platform: one for the sake of demonstration, open for any user but which is not integrated with the neXtProt curation workflow, and one fully integrated in the BioEditor, the neXtProt biocuration tool.

### neXtA^5^-BioEditor architecture and services

**Figure 2.**
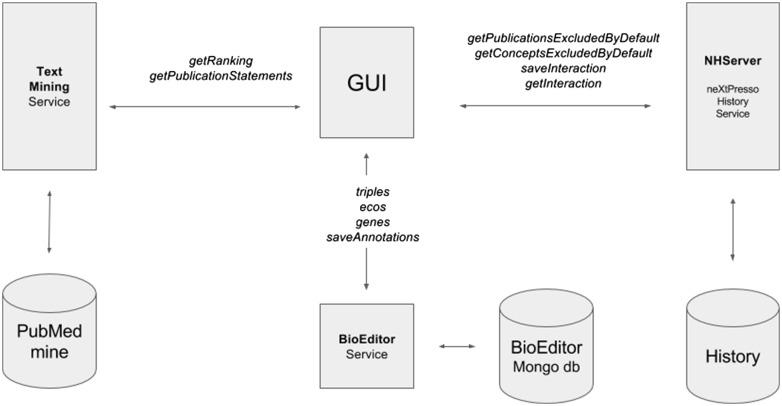
Architecture of the neXtA^5^ implementation in the BioEditor.

As a joint development between SIB Text Mining and CALIPHO, neXtA^5^ attempts to deliver a seamless integration with the curation platform of the neXtProt database, named the BioEditor (27). To achieve this, a set of web services and dedicated user interfaces has been developed. The integration of the neXtA^5^ services within the neXtProt curation workflow is shown in Figure 2, whereas an example of curation using this workflow and a sequencing diagram of the annotation process are, respectively, provided as supplementary material in files 5-a and 5-b.

The two neXtA^5^ services (S3, S4) are server-side programs built with Java REST (Representational State Transfer) technology. These services provide access to the different functionalities: the Information Retrieval models (vector space or Boolean model), the Ranking and the generation of statements for the annotation. Publicly available via URLs, the queries are based on standardized parameters described below.

The service ‘getRanking ’ (S3) is the web service in charge of the classification of documents. Built as http POST request, the output (in JSON or XML format) encloses the publication data as well as the pre-tagged content. For instance, the URL http://candy.hesge.ch/neXtA5/webservice/ranking/json?axis=Disease&gene=BTK would provide the publications relative to the gene ‘BTK’. Other parameters are:

- axis: ‘DISEASE’, ‘GO_MF’, ‘GO_BP’, ‘GO_CC’ or ‘INTERACTION’ depending on the investigated axis, the ranking will depend on this setting.
- mode: should be set to ‘Vectorial’ or ‘Boolean’ depending on the expected research mode.
- synonyms: is a list of gene synonyms, delimited by quotes and separated by commas.
- dateAfter: set at 1980 by default, this is the earliest date of the publications retrieved.
- excludedPublication: is a list of publications’ ID unwanted; these can be already evaluated or declared as non-pertinent by the user.
- excludedConcept: is a list of concepts’ ID unwanted; these are usually considered as non-pertinent by the user.

The service ‘getPublicationStatements’ (S4): operating with http GET method, this service aims to provide the so-called triplets for annotation. This generation of triplets, as an association {subject – relation – object}, is the principal contribution brought to the BioEditor. For instance, we can access the statements proposed for the gene ‘FGFR1’ in the PMID ‘17154279’ via the following URL: http://candy.hesge.ch/neXtA5/webservice/publicationStatements/json?publicationId=17154279&publicationSource=PMID &gene=FGFR1&axis=Disease.

The JSON output is shown in Table 3; the following parameters are required:

**Table 2.** Statistics of testing benchmarks for the different PPIs and PTMs ranking methods

	PPI		PTM	
PUBMED (BASELINE)	0.103	–	0.101	–
BIOMED	0.138	+34.1%	0.158	+57.2%
NEXTA5	0.269	+161.9%	0.280	+178.0%
QUERY REFINEMENT	0.299	+190.7%	0.363	+260.6%

Statistics of testing benchmarks for the different PPIs and PTMs ranking methods. Settings are chosen by trial and errors on the fusion on search scores; the precision between two runs could be affected positively or negatively by the parameters and the weights. Only the best results (at P0) from each engine are displayed here.

**Table 3.** JSON output example of the service S4 related to the generation of triplets for the annotation

{
“**gene**”: “FGFR1”,
“**axis**”: “Disease”,
“**publication**”: “17154279”,
“**annotations**”:
[

{

“**proposedRelation**”:{“id”: “”, “name”: “causes disease”},

“**proposedObject**”:{“id”: “C75479”, “name”: “Kallmann syndrome”},

“**proposedEco**”:{“id”: “”, “name”: “”},

“**passage**”:“In a new cohort of 141 unrelated patients affected by Kallmann syndrome we identified FGFR1 sequence variants in 17 patients, all in the heterozygous state.”

},

…
]
}

JSON output example of the service S4 related to the generation of triplets for the annotation. In this publication, PMID 17154279, the authors report on the possible implication of the gene *FGFR1* in the Kallmann syndrome.

- gene: names the investigated gene.
- axis: designates the same axes as above.
- publicationId: is the pmid from which the user wants to extract findings.
- publicationSource: ‘PMID’ or ‘PMCID’, the first one is selected by default since the full-texts are not ready for a stable release.

Three services were implemented to fetch various configuration settings:

- service ‘genes’ (S0): returns the list of genes available in neXtProt. This list is used to propose all human proteins that can be annotated;
- service ‘triples’ (S0): returns the possible relations and objects for one axis to constrain the user when creating annotations;
- service ‘ecos’ (S0): returns the list of the Evidence and Conclusion Ontology (ECOs) concepts that are allowed to be issued in conjunction with each relation.

Four additional services were implemented to allow the user to filter out results from ‘getRanking’ service and to track user interactions, which later can support an improved user experience:

- service ‘getPublicationExcludedByDefault’ (S1): returns publications already treated for a given doublet gene/axis to allow user to filter displayed publications;
- service ‘getConceptsExcludedByDefault’ (S2): returns already treated concepts [for instance, ‘lactase activity’ (GO:0000016)] for a given doublet gene/axis allowing user to filter displayed publications;
- service ‘saveInteraction’ (S5): stores modifications done by a user for a given triplet publication/gene/axis. For each annotation, it records: the axis, the gene, the publication, the proposed object, the proposed relation, the proposed ECO, the status (accepted, modified, rejected or unreviewed). Then, if the annotation is accepted or modified, the selected object, the selected relationship and the selected ECO descriptor are saved.
- service ‘getInteraction’ (S7): returns the saved interactions for a given triplet publication/gene/axis.

The last implemented service (S6) saves annotations in the BioEditor. It stores all accepted and modified annotations of a given triplet publication/gene/axis.

### neXtA^5^ graphical user interface

**Figure 3.**
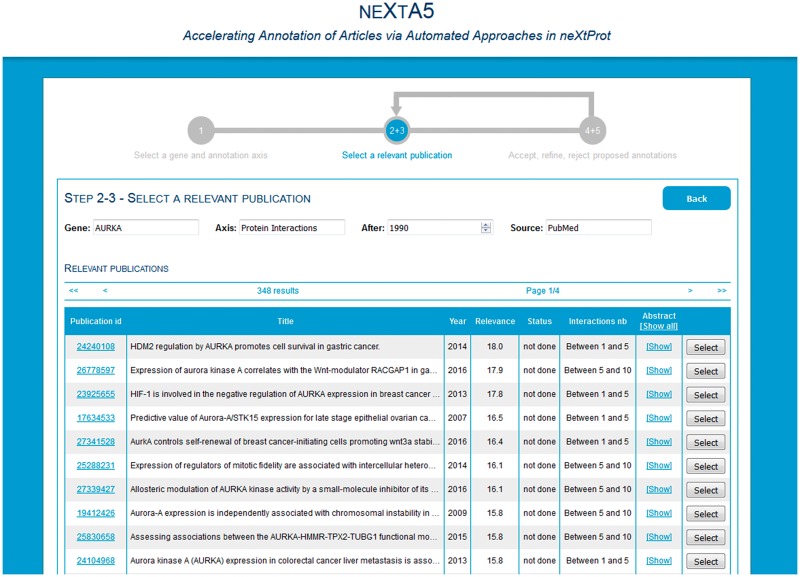
Phase 2, selection of a relevant publication in the ranked list.

**Figure 4.**
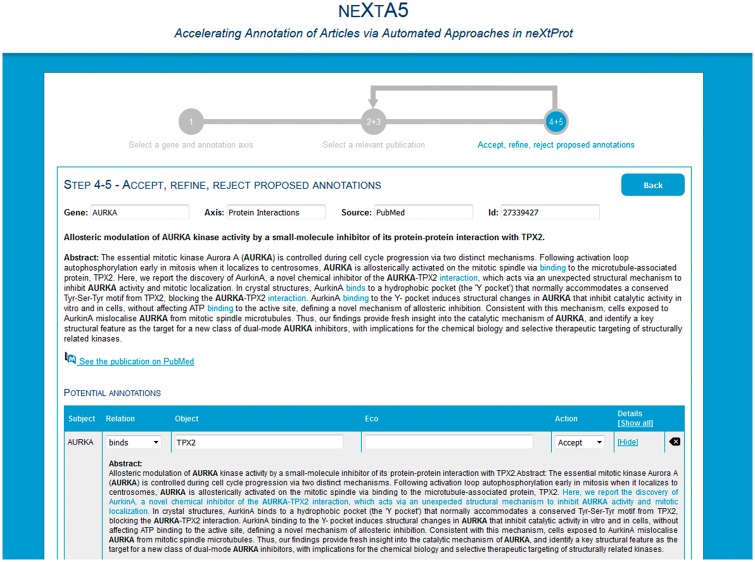
Phase 3, curation of the proposed annotations.

A web interface is available (http://candy.hesge.ch/nextA5) for an open trial of the text-mining resources. The process is split into three steps. First, the user indicates the gene (e.g. FGFR1) and the axis (e.g. diseases) on which s/he would like to work. Immediately, the publications and concepts previously annotated (in the BioEditor) for this gene and axis are retrieved and displayed. The user can select publications and/or concepts to exclude. Second, the system retrieves some publications and displays them in a table (Figure 3). Publications are ranked by relevance. For each publication, the user can view the abstracts with some highlighted concepts, along with the status of the annotation (e.g. partial, not done). Third, the user selects a publication and the system retrieves the suggested annotations (Figure 4). Annotation statements are displayed as triplets, but at the current stage, only the object is automatically proposed. The user must therefore manually complete the triplet (i.e. the relation and the ECO descriptor), with the help of autocomplete functionalities. An annotation can be ‘accepted’, ‘modified’, ‘rejected’ or ‘unreviewed’ (pending). At any point, the user can save his work by clicking on the *Save temp* button and come back later. Once the publication is fully annotated (i.e. all the annotations have been reviewed), the user can save the annotations in the BioEditor by clicking on the *Save* button.

## Conclusion

The linear combination of heterogeneous IR outcomes is a very effective approach to strengthen triage and the current improvements clearly exceed the initial plans: from +191% (PPIs) to +261% (PTMs). The global triage effort can thus potentially be reduced by a factor 3. Although triage of positive and negative articles can represent up to 15% of the curation work, we can significantly speed up this step, which could finally represent <5% of the whole curation process. The overall productivity gain for biocurators would thus be in the range of 10%. The impact on other dimensions of curation (paper reading, normalization of descriptors and reduction of the overall process time) is still to be evaluated.

The general approach of the neXtPresso project, ‘one curation axis-one dedicated search approach’, seems therefore extremely effective. Pushing the approach further, we could envisage, in the future, that different engines could be applied in parallel or as batch processes instead of adopting user-piloted interactive tools. This approach has also the advantage that a virtually infinite list of ranking features can be tested independently to design a better scoring function.

Further improvements to the system may be obtained by using not only positive features, but also negative ones. Indeed, query refinement is well-known approach to perform query reformulation, but more advanced approaches have been proposed by researchers in information sciences. In particular, the Rocchio algorithm showed competitive results in many settings (46). Rocchio is an interactive method in which the end-user manually selects relevant articles out of the list retrieved by the system. By selecting relevant articles, the end-user also implicitly defines non-relevant ones. From this dataset, it is then possible to sort the most frequent words associated with each subset. This list is then shown to the user, who can manually add or remove keywords (47). Negative features are not added to the original queries, but instead they are used to filter out irrelevant publications. Such an approach is intended to reduce information deluge. It is clear that future developments in text mining applied to curation could significantly improve if curation platforms would capture not only papers (and information) they select, but also papers they reject as argued in (22). 

## Funding

This work was supported by the Swiss National Fund for Scientific Research, SNF Grant: [neXtPresso project, SNF #153437]. The open access publishing charge was supported by the University of Applied Sciences, Western Switzerland/HEG Genève/BiTeM group.

*Conflict of interest*: None declared.

## Supplementary Material

Supplementary Data
