# Supplementary material for: Triage by ranking to support the curation of protein interactions
Source: Database (Oxford). 2017 Jun 11;2017:bax040. doi: 10.1093/database/bax040 (PMC5502361; doi:10.1093/database/bax040)
Supplement: Supplementary Data [file bax040_Supp.zip › Supplementary_File_5-a_curation_workflow_using_the_neXtA5-BioEditor_tools.docx]

**Use case: curation workflow using the neXtA5-BioEditor tools**

Case: Send new annotation to the BioEditor

Actor: A curator from the neXtProt group

Context: neXtA^5^ GUI, neXtA^5^ services, BioEditor services

Precondition: An authorized access to the BioEditor

1. Find interactors for the human protein BTK.
2. On the neXtA^5^ interface, the user fulfils the fields “gene” and “axis” with regard to his query.
3. The user validates his choice and the system returns the top 100 relevance-based publications.
4. The user selects a publication and the system returns the complete list of annotations according to the query.
5. For each annotation:
   1. The user asks the system to display the passage relative to the evidence.
   2. The user checks the pertinence of the proposed subject/relation/object/evidence.
   3. The user accepts the annotation.
   4. The user completes blank(s) when necessary.
6. The user saves the work performed on this publication, which is sent to the BioEditor.
7. The user logs in the BioEditor, and finally saves the annotation.

Alternatives

- 1. The user wants to limit his research on the literature post-2000. He selects the year in the drop-down list “published”.
  2. The user has already worked on a few publications. He does not want to see these ones anymore and adds the publications’ ID in the “Excluded publication” field.
  3. The user is not interested in evidences relative to “co-immunoprecipitation”. He adds this term in the “Excluded concept” field.
     1. The user refuses the annotation.
  4. The user temporary saves his work on this publication.
