# Supplementary figures and images for: Triage by ranking to support the curation of protein interactions
Source: Database (Oxford). 2017 Jun 11;2017:bax040. doi: 10.1093/database/bax040 (PMC5502361; doi:10.1093/database/bax040)

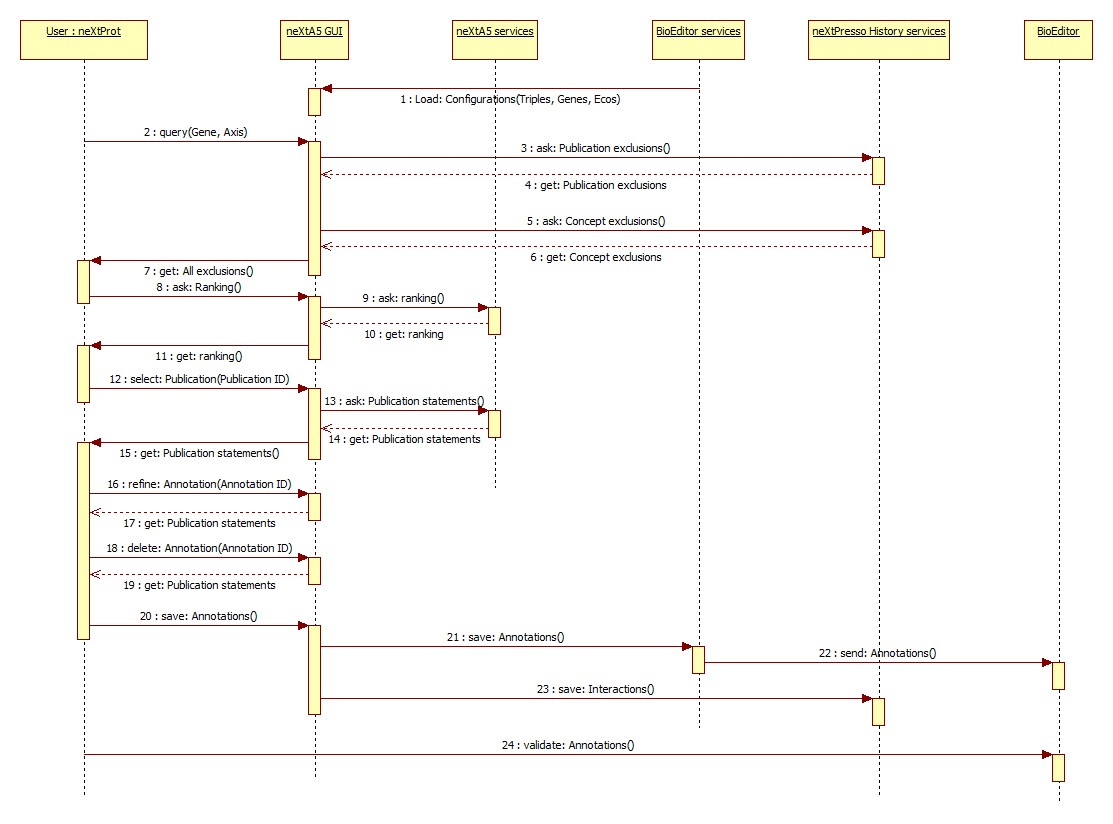

Supplement: Supplementary Data [file bax040_Supp.zip › Supplementary_File_5-b_Sequencing_diagram_of_the_annotation_process.png]
